# Recent advance in phytonanomedicine and mineral nanomedicine delivery system of the treatment for acute myeloid leukemia

**DOI:** 10.1186/s12951-023-01968-2

**Published:** 2023-07-26

**Authors:** Yimin Jia, Cun Sun, Ting Chen, Hui Zhu, Tianrui Wang, Yan Ye, Xing Luo, Xiaoqiang Zeng, Yun Yang, Hao Zeng, Quanming Zou, Enqiang Liu, Jieping Li, Hongwu Sun

**Affiliations:** 1 Chongqing University Cancer Hospital, Chongqing, 400030 China; 2 Department of Microbiology and Biochemical Pharmacy, College of Pharmacy, Third Military Medical University, Chongqing, 400038 China; 3 Department of Hematology and Oncology, Qianjiang Central Hospital of Chongqing Municipality, Qian Jiang, Chonqing, 409000 China

**Keywords:** Acute myeloid leukemia, Phytomedicine, Mineral medicine, Drug delivery system, Nanomedicine

## Abstract

Acute myeloid leukemia (AML) is an invasive hematopoietic malignancy caused by excessive proliferation of myeloblasts. Classical chemotherapies and cell transplantation therapies have remarkable efficacy in AML treatment; however, 30–40% of patients relapsed or had refractory disease. The resistance of AML is closely related to its inherent cytogenetics or various gene mutations. Recently, phytonanomedicine are found to be effective against resistant AML cells and have become a research focus for nanotechnology development to improve their properties, such as increasing solubility, improving absorption, enhancing bioavailability, and maintaining sustained release and targeting. These novel phytonanomedicine and mineral nanomedicine, including nanocrystals, nanoemulsion, nanoparticles, nanoliposome, and nanomicelles, offer many advantages, such as flexible dosages or forms, multiple routes of administration, and curative effects. Therefore, we reviewed the application and progress of phytomedicine in AML treatment and discussed the limitations and future prospects. This review may provide a solid reference to guide future research on AML treatment.

## Introduction

Leukemia, including acute myeloid leukemia (AML), chronic myeloid leukemia, acute lymphoblastic leukemia and chronic lymphocytic leukemia, is a life-threatening malignant disease characterized by the uncontrolled proliferation of mutant progenitor cells and inhibition of normal hematopoiesis [1]. As an aggressive blood malignancy, AML is marked by the accumulation of immature blood cells, which severely interferes with the normal hematopoietic system. The 5-year survival rate is approximately 30%; therefore, developing novel targeted therapies or treatments has become an important research focus [2–4]. Global Burden Disease reported that the incidence of AML increased from 63,840 to in 1990 to 119,570 in 2017 [5]. It is the most common form of leukemia and accounts for approximately 25% of all leukemia cases in adults in Western countries. Moreover, the incidence increases dramatically with age, from 1.8 cases per 100,000 people younger than 65 to 13.7 cases per 100,000 people older than 65[6].

### AML development

Current AML treatments include chemotherapy, radiotherapy and immunotherapy. In addition to disease recurrence, treatment-related toxicity, especially from chemotherapy, is associated with poor prognosis [7]. The treatment of AML is complex, with multiple cycles of combined cytosine arabinosine (cytarabine)- and anthracycline-containing chemotherapy regimens and the selection of eligible patients for autologous or allogeneic stem cell transplantation. For example, chemotherapy achieves a complete remission rate of 60–85% in patients older than 65 years of age. However, 60% of patients subsequently relapse, which results in a 5-year overall survival of 40%. Because of poor cytogenetic risk, the overall survival drops to 10% in older patients [8]. Therefore, finding an effective treatment is important for the control of AML.

### AML chemical drug treatment

After common treatment within 1 year of diagnosis, many young patients can achieve remarkable control, but up to 70% of patients older than 65 years will die [9]. In addition, timely therapy for AML is essential, especially when tumor lysis syndrome or disseminated intravascular coagulation strings occur along with the rapid proliferation of malignant blasts [10]. Common chemical drugs for AML treatment include daunorubicin, cytarabine, azacitidine, trans retinoic acid, and decitabine. The most effective therapeutic component, cytarabine, was discovered and combined with other drugs to produce the first treatment regimen [11]. Cytarabine, a structural analog of deoxycytidine, is one of several nucleosides isolated from sponges and first synthesized in 1959. It can achieve between 25% and 45% remission, while combination treatment with anthracycline can achieve from 50–80%[12–15]. In low-dose regimens, the main adverse effects of cytarabine are limited to the gastrointestinal tract and bone marrow. Recently, there have been many reports on the neurotoxicity of cytarabine in typical and high-dose regimens, including relatively mild peripheral nervous system syndrome and acute, sometimes serious cerebellar toxicity [16]. Therefore, a protocol composed of 100 to 200 mg/m^2^ cytarabine for seven days of continuous infusion and 45 to 60 mg/m^2^ daunorubicin daily for three days of intravenous infusion has been the typical scheme for several decades. This classical “3 + 7” regimen can achieve a curative ratio of 30% in generally younger patients and of 10-15% in older people [17]. Although it has become the gold standard for treatment, its high toxicity and lack of ideal efficacy have made the development of effective and safe drugs for AML a topic of intense interest.

### AML Relapse

Importantly, relapse of AML remains a significant challenge [18]. The non-reactive or relapse rates are still approximately 75–80% after the front-line treatment regimen of AML [19]. It is still a common scenario in further treatment, and the occurrence is 40–50% in younger patients and a large proportion of elderly patients [20]. Thus, relapsed/refractory AML remains a serious challenge in the clinic [21]. Approximately 25–30% of relapsed AML patients are resistant to ATRA (all-trans retinoic acid) treatment. Although targeted inhibitory drugs or antibodies, such as IDH1/IDH2, FLT3, CD33, CD123 and BCL2, can greatly prevent relapse and improve the efficacy of treatment for AML [22], new mutation points are rapidly and continuously emerging because of drug resistance and the likelihood of relapse. Moreover, the side effects of chemotherapy drugs are often unbearable, leading to many complications and poor quality of life. Therefore, it is urgent to look for a more ideal treatment to control AML relapse.

### AML phytomedicine and mineral medicine

**Fig. 1:**
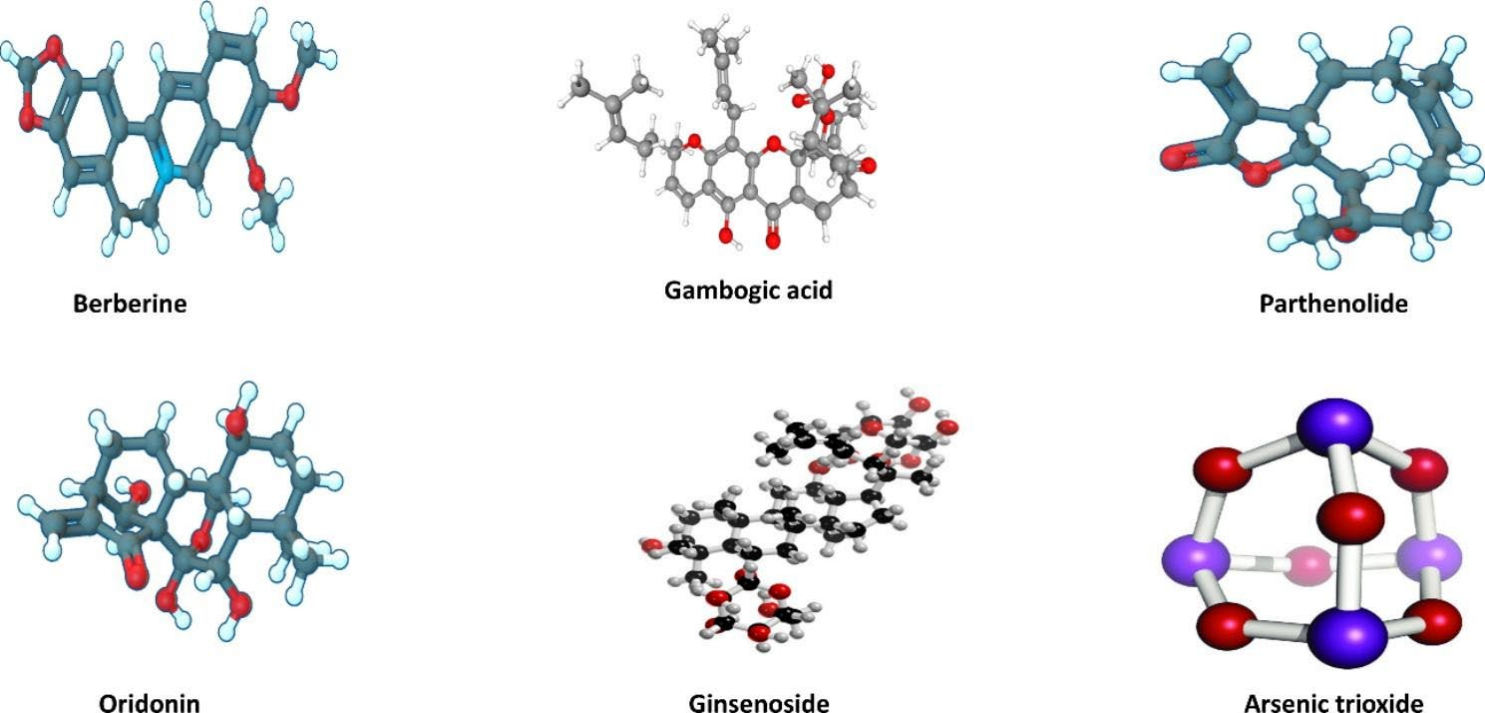
Phytomedicine and mineral medicine of AML treatment **Note**: It is well known that mineral containing arsenic and its derivatives (such as arsenic trioxide and arsenic tetrasulfide), phytomedicine including berberine, gambogic acid, parthenolide, oridonin and ginsenoside have been applied in AML treatment

It has been found that a few active ingredients in phytomedicine and mineral can significantly improve the treatment efficacy, especially for relapsed AML [23–25]. Controlled-release phytomedicine has attracted great attention for AML released and treatment, because controlled release drugs have many advantages such as safety, effectiveness and reduction of drug resistance. There is a great deal of medicine, such as camptothecin, paclitaxel, podophyllotoxin, vinblastine, and vincristine, in the market [26]. It is well known that mineral containing arsenic and its derivatives (such as arsenic trioxide and arsenic tetrasulfide), phytomedicine including berberine, gambogic acid, parthenolide, ordonin, grinsenside, and rubesidin have been applied in AML treatment in the Fig. 1. Arsenic and its derivatives have been used for more than 3,000 years [27]. It exists in various forms; most of the time, it is combined with metals as well as nonmetals such as oxygen and sulfur. White arsenic or arsenic trioxide (As_2_O_3_) is obtained by conventional heating [27]. As early as the 1970s, researchers at Harbin University began using it to treat various malignant and AML tumors [28]. However, the insolubility, high potential toxicity and low bioavailability of arsenic-containing mineral medicine are shortcomings of some therapeutic plant ingredients. However, the further development of modern nanomedicine delivery systems, such as nanoliposome [25], microcapsules [29], nanoemulsion [4], solid dispersions [30], and magnetic nanoparticles [30], to address this problem could be highly beneficial. Nanotechnologies could improve efficacy, increase drug stability, and provide greater flexibility of form and multiple drug delivery routes for Traditional Chinese medicine [31]. Their advantages include the ability to realize sustained release, increase the biological half-life, and prolong the circulation time in the body [32, 33]. Since phytomedicine has been useful in treating epidemic diseases for a long time in Chinese history, the accumulated experience has led to success for AML treatment in terms of alleviating clinical symptoms, increasing the cure rate of disease, and hindering disease progression [3]. Many studies on the nanomedicine of phytomedicine and mineral have been applied in AML treatment. Therefore, this review discusses the pharmacological effects, pharmacokinetics, and cytotoxicity of the nanomedicine delivery systems including phytomedicine and mineral for AML treatment, the modulation of their properties, and the prospects for further research.

## Nanomedicine delivery systems of mineral and phytomedicine

**Fig. 2:**
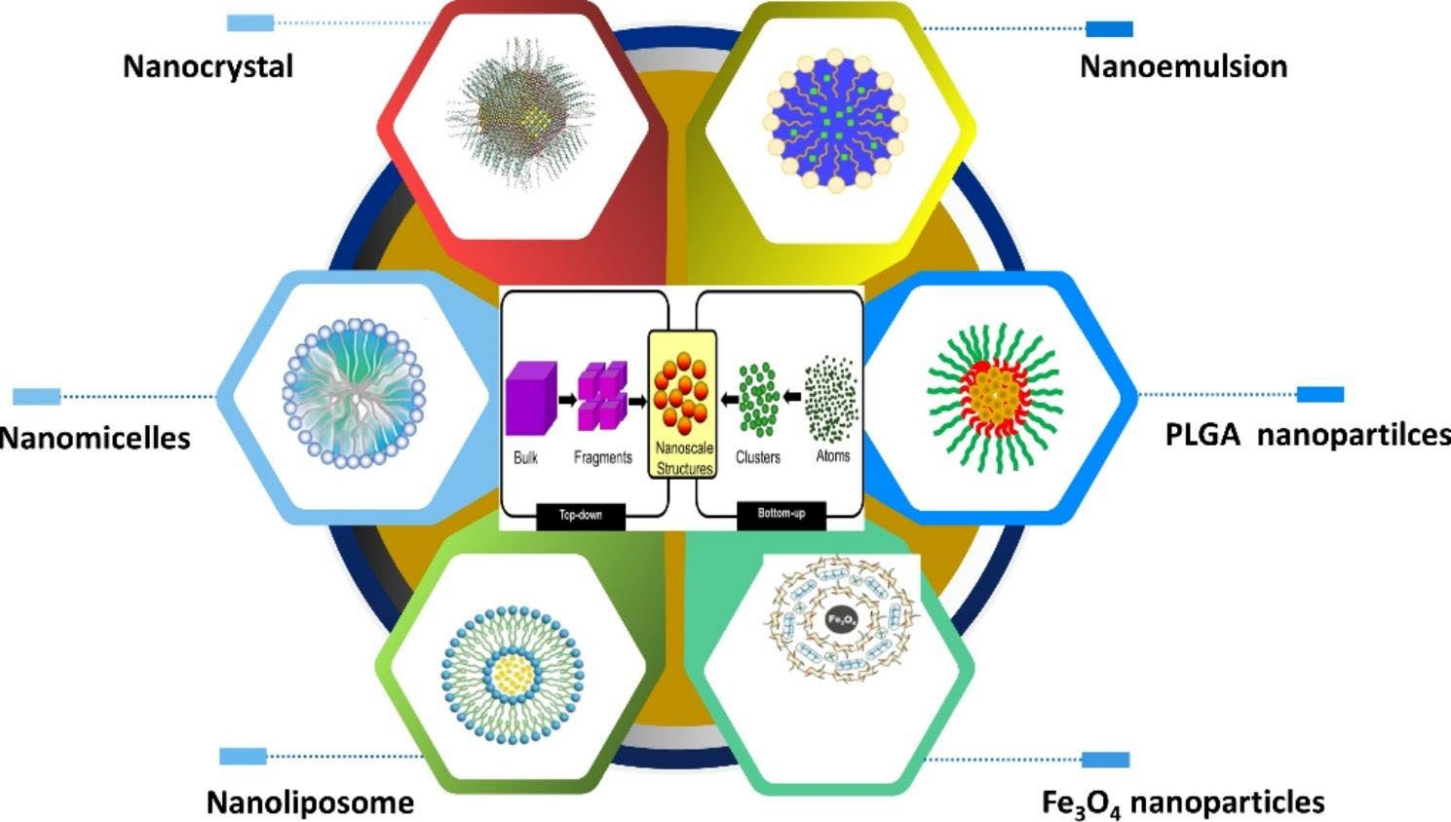
Various nanomedicine delivery systems of AML treatment **Note**: There are variously nanomedicine delivery system of phytomedicine and mineral (Fig. 2) such as nanocrystal, nanoemulsion, PLGA nanoparticles, Fe_3_O_4_ nanoparticles, nanoliposome and nanomicelles in the AML treatment

It is well known that nanomedicine, the application of nanotechnology to the treatment diseases, has great promise in the treatment of cancer, viral infections and other fatal diseases. Recently, several nanomedicine have been reported, including liposome products already on the market, such as Myocet™, Doxil®, Onivyde™, Caelyx®, Abraxane® and Vyxeos®^[34]^. There are variously nanomedicine delivery greatsystems of phytomedicine and mineral (Fig. 2) such as nanocrystal, nanoemulsion, PLGA nanoparticles, Fe_3_O_4_ nanoparticles, nanolipomses and nanomicelles in the AML treatment. These nanomedicine are making momentous progress by overcoming the limitations of traditional drugs and meeting unmet clinical needs [32]. Moreover, it has recently gained prominence due to the ability to engineer its structural morphology to ensure targeted and controlled release. Additionally, nanomedicine can be optimized to enter target cells, an advantage of their comparatively small particle size. Nanomedicine also have the advantages of high drug loading rates and targeting ability, which can prevent effects on healthy tissues [33]. In retrospect, nanomaterials had a long history of medical use during ancient times. The Bhasma (nano-metal ash) which was included in Ayurveda to treat a variety of diseases by Indians since the 7th century was a kind of nanomaterials [26].

### Nanocrystals

**Fig. 3:**
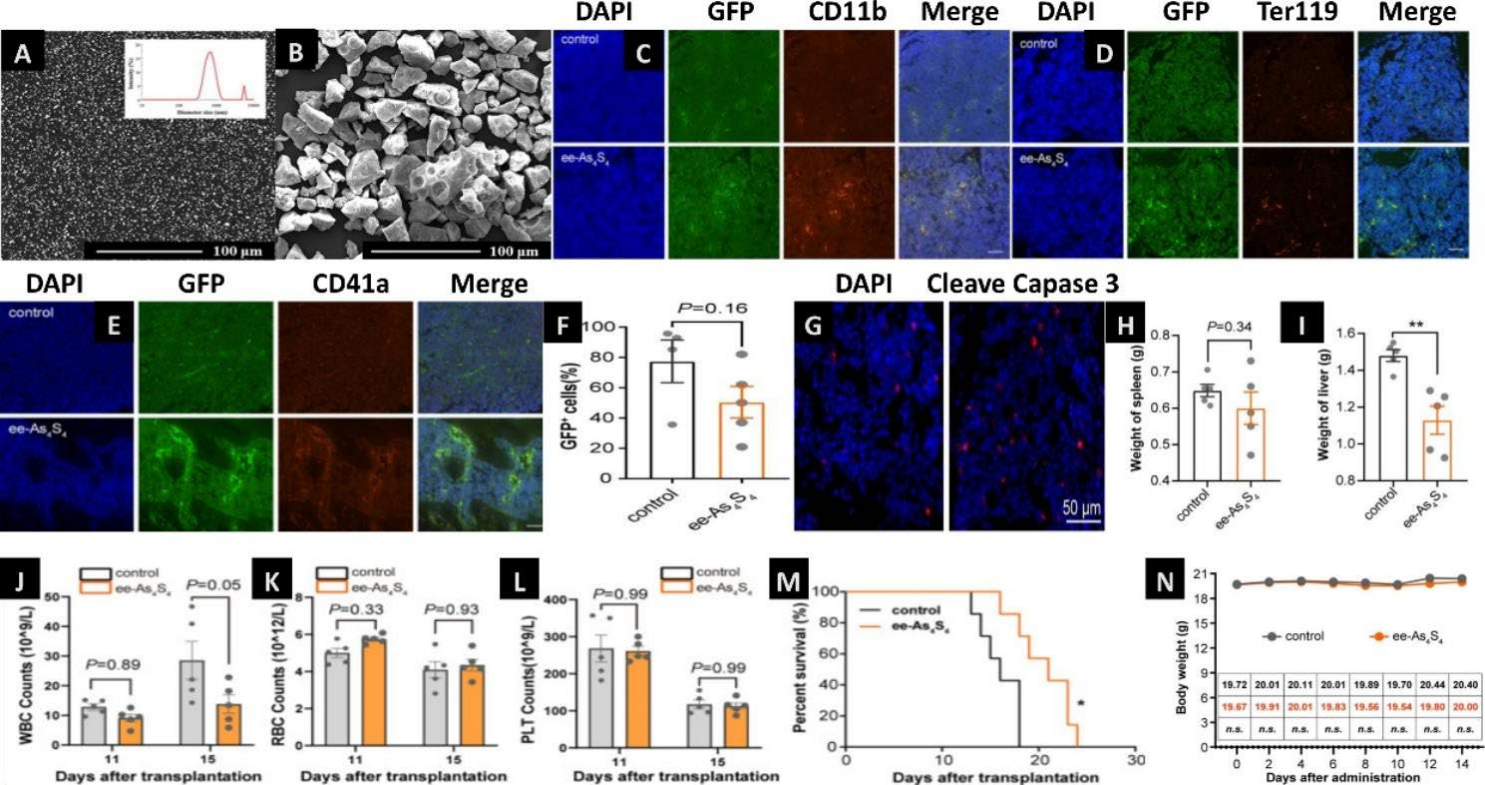
Nanocrystal delivery system of AML treatment **Note**: SEM images of realgar particles in e-As4S4 (**A**) and r-As4S4 (**B**). (Figure source from the reference [41], color figure can be viewed at nature.com). (**C-E**) Immunofluorescence staining images of three markers (CD11b, Ter 119 and CD41a) in femurs of mice after treated with ee-As4S4. Scale bar indicates 100 μm. (**F**) The percentage of GFP^+^ leukemia cells in the bone marrow determined by flow cytometry. (**G**) Immunofluorescence staining of cleaved caspase-3 in the femur of mice from control and ee-As4S4 group. (**H** and **I**) The spleen and liver weight of AML mice (n = 5). (**J–L**)The counts of white blood cell (WBC), red blood cell (RBC), and platelet (PLT)(n = 5). (**M**) Survival curve of AML mice (n = 7). *P < 0.05,**P < 0.01. (**N**) The body weight in vivo safety of ee-As4S4 in mice(n = 6).(Figure source from the reference [42], color figure can be viewed at dove press.com)

Nanocrystal drugs with sizes in the nanometer range are prepared by using drugs as the sole component [34], and the solubility and dissolution rate are greatly improved [35]. The particle size of realgar arsenic powder decreases with increasing grinding time of the ball mill. A smaller particle size (330 nm) is obtained at 6 and 12 h, and the polydispersity index of the sample is approximately 0.3. Ye Hanqing dispersed As_2_O_3_ in realgar solid with an average particle size of approximately 200 nm. These particles can significantly inhibit the growth of HL-60 cells, by causing ROS production, lipid peroxidation, and protein oxidation, especially the oxidation of reduced sulfhydryl groups, destroying the balance of the intracellular antioxidant enzyme system and changing the oxidative reduction level of glutathione in cells. Additionally, it regulates the level of transcription and/or translation rather than the level of mRNA. It can stimulate the activities of Mn SOD and GPx antioxidant enzymes [36]. Superfine grinding is another novel technique widely applied to metal materials and drugs to prepare superfine powder with good surface properties(polydispersity and solubility)[37]. In Wang Chuan’s study, a realgar water-dispersible solid material (E-ASASS4) was prepared by mixing the polymer matrix compound with raw R-ASASAS4 using hot melt extrusion technology. The dissolution and bioavailability of the novel nanomaterials were increased by 204 and 12.6 times, respectively. Additionally, it was found that E-AS_4_SA had a 4.4 times higher ability to inhibit cell proliferation in HL-60 cells than R-AS4SA. These data suggested that E-AS_4_SA can effectively improve the therapeutic effect, inhibit the proliferation of leukemia cells, reduce the occurrence of liver, spleen and extracellular infiltration, and prolong the survival time of mice [38]. Another study suggested that at a very low concentration (3.9 mg/L), these novel nanoparticles with particle sizes of 23.8 nm, 171 nm, and 167 nm could kill approximately 80% of tumor cells. Moreover, these nanoparticles blocked S-phase cells and G0/G1-phase cells. They reported that the average diameter of the e-As4S4 (680 nm, Fig. 3A) was significantly smaller than r-As4S4 (28.9 μm, Fig. 3B)[39]. Also they found that the nanocrystal ee-As4S4 increased the expression of CD11b, Ter119 and CD41 in leukemic cells isolated from mouse bone marrow (Fig. 3C and E).They found also that the percentage of GFP^+^ cells in the bone marrow decreased from 77.4% in the control group to 53.0% with ee-As4S4 treatment (Fig. 3F). Furthermore, caspase-3 was increased in the femur of ee-As4S4 treated mice, indicating that it induced significant apoptosis in vivo (Fig. 3G). Leukemia cell infiltration was relieved received ee-As4S4 treatment as the spleen weight was slightly reduced (Fig. 3H). The average weight of the livers was reduced to 1.13 g in the ee-As4S4 group, while that was 1.48 g in the control group (Fig. 3I). The peripheral white blood cell was significantly reduced by ee-As4S4 administration in the mice, suggesting the remission of AML (Fig. 3J). The red blood cell in the ee-As4S4 group can reveal an anemia-alleviating effect (Fig. 3K). However, it did not affect the platelet count (Fig. 3L). What’s more important, the novel nanocrystal can prolong the survival of mice compared with control group (Fig. 3M). In vivo safety assessment results showed that there were no significant difference in the bodyweight for both groups (Fig. 3N). These data suggest that this nanocrystal may induce effective and multiple-lineage cell differentiation and apoptosis in the refractory AML mouse model and cell line, suggesting that it holds promising potential as a novel inductive agent in AML therapy [40].

### Nanoemulsion

**Fig. 4:**
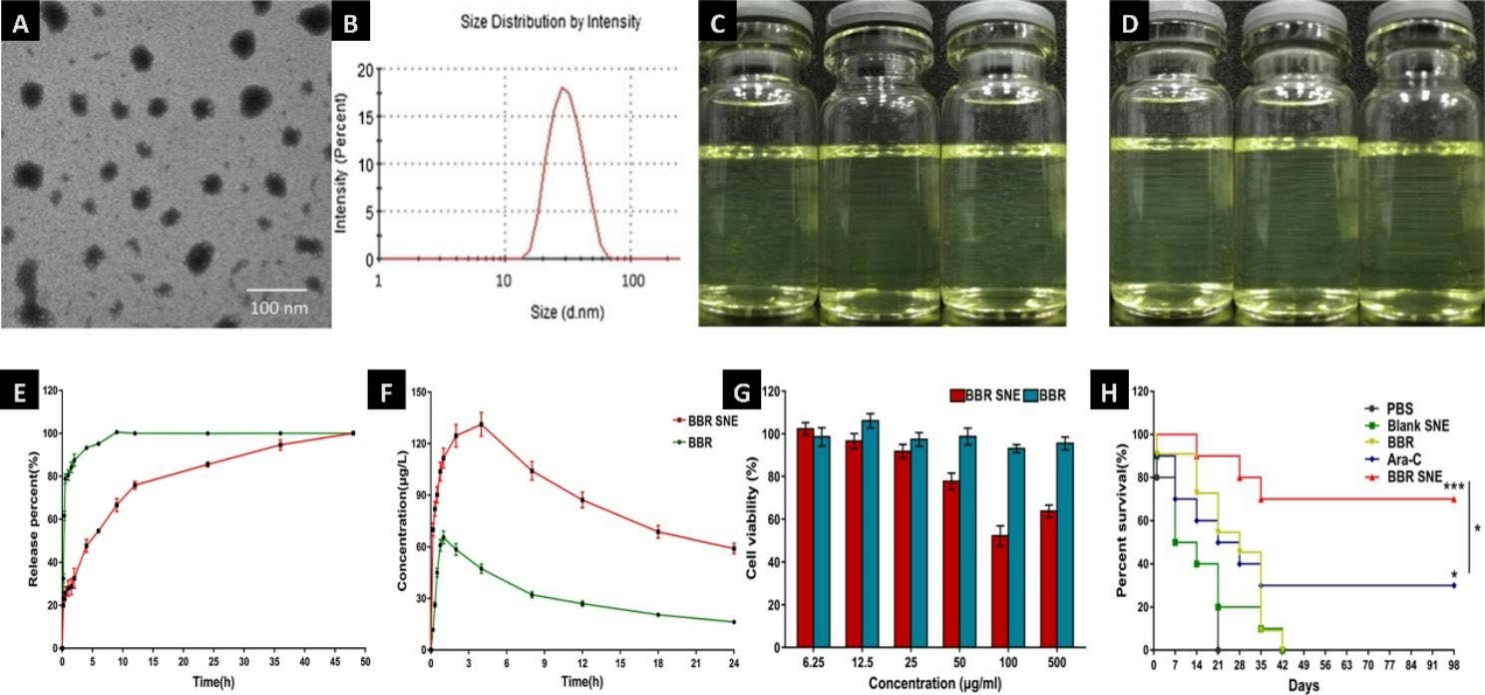
Nanoemulsion delivery system of AML treatment **Note**: Morphological (**A**) and size distribution (**B**) of berberine nanoemulsion (BBR NE). Storage stability assessed by storing in ambient conditions for 30 days: changes of appearance vs. time at 25 °C (**C**) and 37 °C (**D**). (Figure source from the reference [46], color figure can be viewed at elsiver.com). In vitro profiles of BBR NE in the (**E**) In vitro release profle of the berberine suspension (BBR) and the system (BBR SNE) in PBS (pH = 7.4) (n = 3), (**F**) Plasma concentration profles of berberine after oral administration of BBR and BBR SNE in healthy rabbits (n = 6) (**G**) Effect of berberine solution (BBR) and the system (BBR SNE) on Caco-2 cell viability as evaluated by MTT assays after treatment for 24 h. The data are expressed as the mean ± S.D. (n = 3) (**H**). The survival rate of MV4-11 xenograft mice orally administered berberine suspension (BBR), PBS, Blank SNE and the system (BBR SNE) excepted for cytarabine (Ara-C) with the vein intravenous injection at 50 (mg/kg)/day for 14 consecutive days; the same volume of PBS and blank SNE were used as controls. The data are expressed as the mean ± S.D. (n = 10); *P < 0.05, and ***P < 0.001. (Figure source from the reference [44], color figure can be viewed at nature.com)

Nanoemulsion with homogeneous size distributions and sizes from 1 to 100 nm are stable, isotropic, transparent or translucent systems composed of water, oil, surfactants, co-surfactants, etc [43, 44]. They are not subject to coalescence, flocculation or sedimentation and are therefore stable carriers [45, 46]. In recent years, some phytomedicine, including gambogic acid and berberine, have been used in nanoemulsion drug delivery systems to improve poor solubility and instability caused by temperature and light [47]. For example, the gambogic acid nanoemulsion may improve the survival time and survival rate of mice engrafted with MV4-11, which implies a much better pharmacological effect against AML in vivo and in vitro [48]. Other phytomedicines, such as berberine, have many disadvantages, such as low water solubility and low bioavailability (1%); high excretion rates in the intestine, liver and gallbladder; multidrug resistance resulting from the main risk of being pumped out by P-gp; and poor intestinal membrane permeability [49]. Researcher found that spherical droplets of berberine nanoemulsion (BBR NE) are observed and the droplet size is about 30 nm (Fig. 4A and B). Moreover, there is no phase separation and drug precipitation when stored at 25℃ (Fig. 4C) and 37℃ (Fig. 4D) within one month. The near complete release time of a self nanoemulsifying system was delayed 14.4-fold compared with that of the suspension of berberine (Fig. 4E). Te AUC of this self nanoemulsifying system (3639.94 ± 10.37 µg/mL h) was 3.41-fold greater than that of the BBR suspension (1071.26 ± 6.15 µg/mL h) (Fig. 4F). The BBR self nanoemulsifying system showed no significant cytotoxicity (cell viability > 90%) at concentrations between 6.25 and 500 µg/mL (Fig. 4G)[49].The self-nanoemulsifying systems can avoid first-pass metabolism to enhance the drug absorption rate and improve membrane transport speed and solubility. Hence, scientists have applied the phase inversion preparation method to prepare a berberine self-nanoemulsifying system (BBR SNE) consisting of 0.5% BBR, 2.4% 1,2-propanediol, 9.6% RH-40 and 0.3% squalene. This drug delivery system had a comparatively homogeneous particle size distribution (PdI = 0.121 ± 0.01, less than 0.3) and good stability potential (4.17 ± 0.82 mV). Compared to mice treated with BBR or blank SNE (no survival), the mice treated with BBR SNE exhibited a higher survival rate of 70% at 98 days, which suggested that this unique system observably improved the overall survival of mice engrafted with MV4-11(Fig. 4H)[44].

### Nanoparticles

**Fig. 5:**
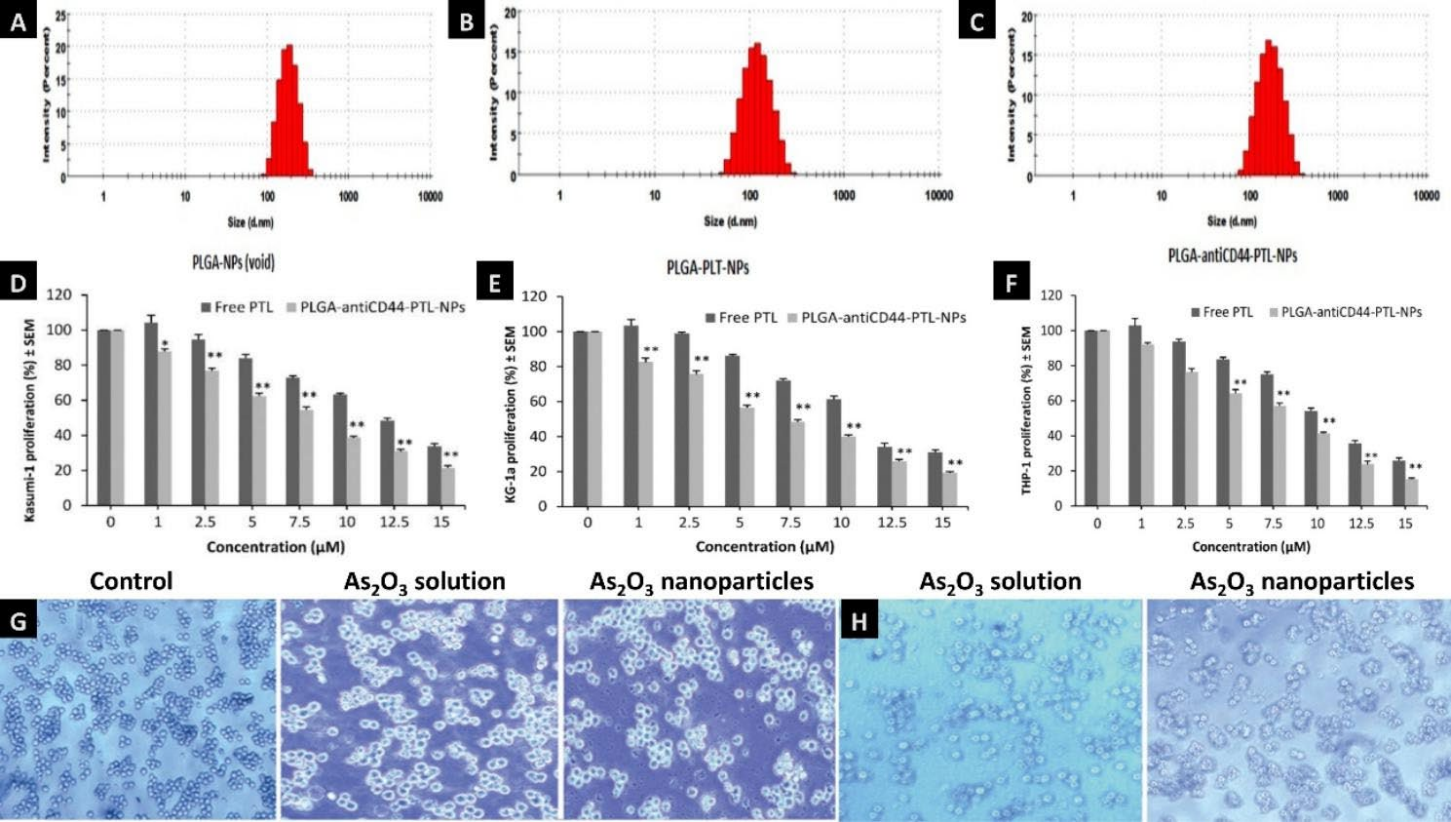
Nanoparticle delivery system of AML treatment **Note**: (**A-C**). Size distribution of three kinds of nanoparticles including void PLGA nanoparticles (PLGA-NPs), PLGA nanoparticles encapsulating parthenolide (PLGA-PTL-NPs), PLGA nanoparticles with anti-CD44 and encapsulating parthenolide (PLGA-antiCD44-PTL-NPs). Cell proliferation after treated with free PTL and PLGA-antiCD44-PTL-NPs against (**D**) Kasumi-1, (**E**) KG-1a and (**F**) THP-1 cell lines. Cell proliferation is expressed as mean ± S.E.M (n = 3). One-way ANOVA was used followed by the Newman-Keuls post-test (*P < 0.05, **P < 0.01 compared to PTL). (Figure source from the reference [59], color figure can be viewed at mdpi.com). Optical inverted microscopy images of NB4 cells incubated with As_2_O_3_ solution or its nanoparticles for 48 h (magnification, ×40) at the concentrations of 1.5 µmol/L (**G**) and 3.0 µmol/L (**H**). (Figure source from the reference [53], color figure can be viewed at spandidos-publications.com)

Nanoparticles within the size range from 1 to 1000 nm have attracted widespread attention in cancer therapies and have promise for AML treatment because they can enhance permeability, reduce adverse effects, prevent multidrug resistance, improve bioavailability, and prolong retention effects with drug circulation [26, 50, 51]. The first nanoparticles drug approved by the FDA was AmBisome®[48]. It has the following advantages: (1) the encapsulation of unstable organic molecules (e.g., nucleic acids and proteins) for protection against degradation; (2) improved stability; (3) surfaces modified with affinity ligands; and (4) controlled release from the materials into a specific site in the body [49]. For example, diterpenoid monomer triptolide (TP) is a natural product of *Tripterygium wilfordii* Hook that has multiple biological activities, but its poor solubility and severe toxic effects on vital organs of the liver, kidney and spleen limit its use in clinical treatment. Therefore, Bing Z Carter found that triptolide nanoparticles at diameters of 100 nm or smaller can effectively induce apoptosis of AML blasts. Other studies suggested that TP nanoparticles can exert a curative effect by significantly increasing the level of ROS in cancer cells, but ROS can also cause lipid peroxidation in metabolic organs such as the liver and kidney and damage normal cell DNA. Additionally, Mei et al. found that the hepatotoxicity of TP-loaded SLN nanoparticles was greatly reduced and the efficacy of AML treatment significantly improved compared to that of the free drug [52]. Xue et al. also found that nanoparticles also improved therapeutic effect by increasing drug absorption and enabling controlled release [53, 54]. Additionally, they found that this novel delivery system has broad drug-loading adaptability and is suitable for multiple methods of administration. Moreover, the particle size and characteristics of these nanoparticles will affect the metabolism and excretion process after oral administration [54, 55]. Recently, it was reported that the cytotoxicity of As_2_O_3_ at concentrations ranging from 0.125 to 1 mg/L to leukemia cancer cells was greatly increased in the leukemia cell line K562 in vitro with dose-dependent inhibition [56]. There are three kinds of nanoparticles including the blank nanoparticles, PLGA-PTL-NPs and PLGA-antiCD44-PTL-NPs were developed. They obtained that size of these nanoparticles were 176, 147 and 162 nm, in the Fig. 5A C. After treatment for 48 h, they found that Kasumi-1 (Fig. 5D) and KG-1a (Fig. 5E) showed 13% (*P* < 0.05) and 17% (*P* < 0.01) decrease in proliferation with 1µM PLGA -antiCD44-PTL-NPs in comparison to free PTL. Moreover, 5 µM PLGA-anti CD44-PTL-NPs were associated with a 35% (*P* < 0.01) decrease in THP-1 cell proliferation (Fig. 5F)[57]. There reported that the NB4 cells were treated with the nanoparticles of As_2_O_3_ in Fig. 5H (3.0 µmol/L) suggested that these cells exhibited a normal shape with similar sizes and clear edges, compared with the cells with As_2_O_3_ solution in Fig. 5G (1.5 µmol/L) were found to be reduced in number and volume and to exhibit irregular shapes [53]. Another reported, the average tumor weight of mice with preexisting tumors after treatment with HHT-MNP-Fe_3_O_4_ was significantly lower than that of mice treated with homoharringtonine alone (193 ± 26 mm^3^ vs. 457 ± 100 mm^3^, *P* < 0.05) and enhanced the inhibit effect on AML cells by inducing apoptosis through the caspase-3 and PARP pathways [58].

### Nanoliposomes

**Fig. 6:**
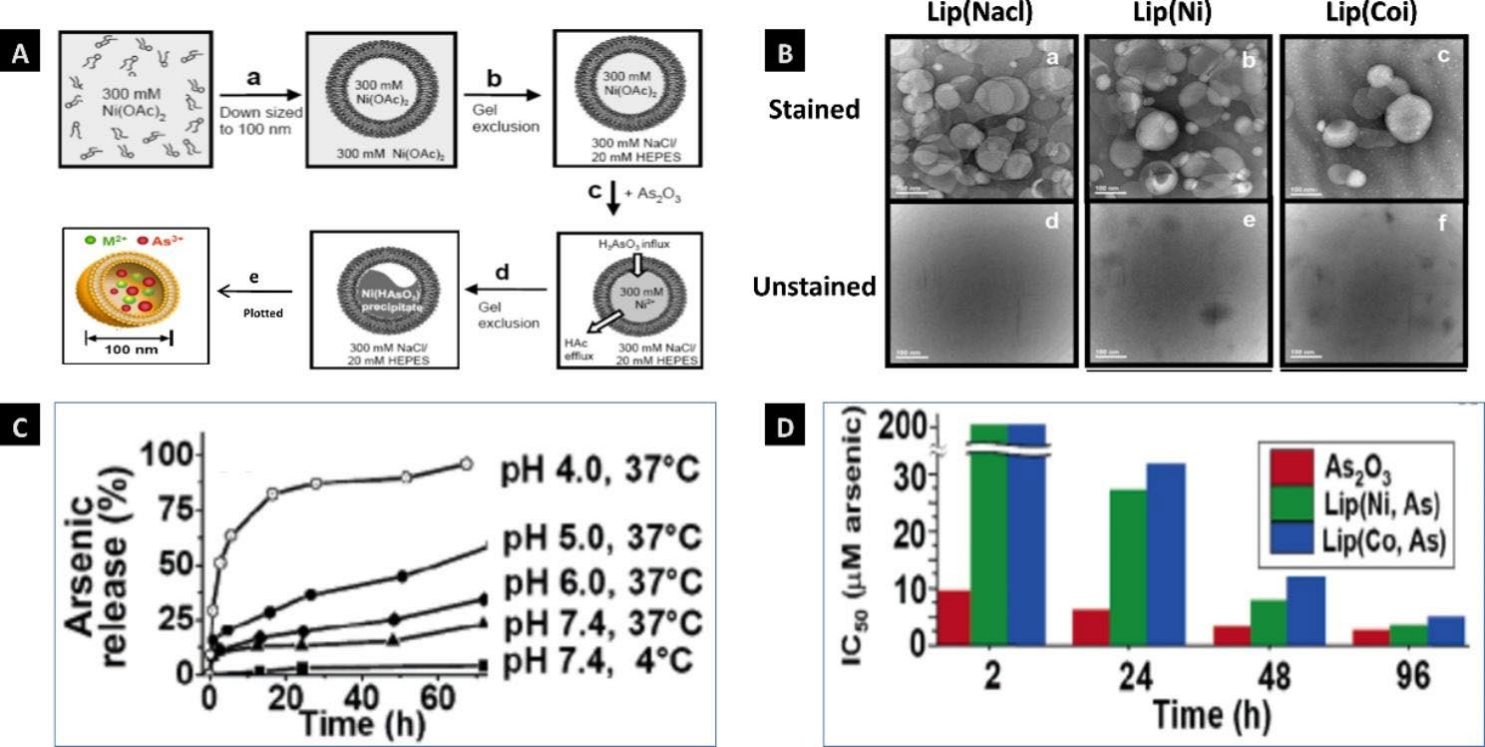
Nanoliposome delivery system of AML treatment. **Note**: (**A**) The procedure of loading arsenic into liposomes, (A-a) Hydration of dried lipids, (A-b) Gel exclusion chromatography.(A-c) Addition of As_2_O_3_ solution into liposomes,(A-d) Influx of H_3_AsO_3_ into liposome, (A-e) plotted of the novel nano-liposomes. (**B**) TEM images of liposomes encapsulating a solution of pH 7.4 (Lip(NaCl), B-a and B-d), pH 7.1 (Lip(Ni), B-b and B-e), pH 7.2 (Lip(Co), B-c and B-f), respectively. These samples of a, b and c were stained with 4% uranyl acetate; the samples of d, e and f were unstained and the micrographs show discrete electron-dense inorganic cores within the liposomes. (**C**) Temperature and pH-triggered arsenic release from Lip-(Ni, As). (**D**) Cytotoxicity of free As_2_O_3_, Lip(Ni, As), and Lip(Co, As). (Figure source from the reference [58], color figure can be viewed at acs.org.com.)

Liposomes with a self-assembling phospholipid delivery system [60, 61] have excellent biological properties, such as biodegradability, biocompatibility, amphiphilicity, low toxicity, a nonionic nature, slow release and active targeting, which provide many opportunities for the delivery of phytomedicines for AML treatment [57]. Importantly, nanoliposome can improve the dispersion, physical and chemical stability and bioavailability of many medicine [62]. The arsenic-loaded liposomes were prepared according to the procedure (Fig. 6A). The accumulation of these liposomes(Lip(Nacl), Lip(Ni), and Lip(Co) could be visualized by transmission electron microscopy (Fig. 6B). They also found that the arsenic drug is stably entrapped under storage conditions (i.e., it exhibits shelf lives > 6 months at 4 °C and pH 7.4, Fig. 6C). As shown in the Fig. 6D, the IC50 values (> 200 µM) of Lip(Ni,As) and Lip(Co,As) were much greater than that of free As_2_O_3_ (9.7 µM)[63]. Moreover, the IC_50_ revealed that both Lip(Ni,As) (IC_50_ = 6.0 µM) and arsenic-loaded liposomes Lip(Co,As) (IC_50_ = 9.6 µM) showed the desired effects of arsenic but lower toxicities than free As_2_O_3_ (IC_50_ = 3.0 µM) after 48 h of treatment of SU-DHL-4 human lymphoma cells. Additionally, the toxicity of this liposome increased markedly to that of free As_2_O_3_, which was consistent with the gradual release of drugs after more than 48 h of incubation [64]. Therefore, these data suggest that free As_2_O_3_ is highly permeable to cell membranes, while these nanoliposome bilayer particles could reduce the acute toxic effects of inorganic arsenic [63]. Research has also shown that nanoliposome delivery has the potential to effectively treat the heterogeneity of AML by targeting the sphingolipid metabolism of ceramide and vinblastine [65]. Ginsenoside (Rg3) is a natural antineoplastic monomer drug extracted from ginseng that can improve the sensitivity of tumors in combination with chemotherapy drugs. A novel biomimetic nanoliposome system (DR@PLip) composed of PM coated on DOX/Rg3 liposomes was developed to treat AML. This system was found to promote the recognition and binding of nanosystems to AML cells through the target interaction with *P*-select in (CD62p)/CD44[66].

### Nanomicelles

**Fig. 7:**
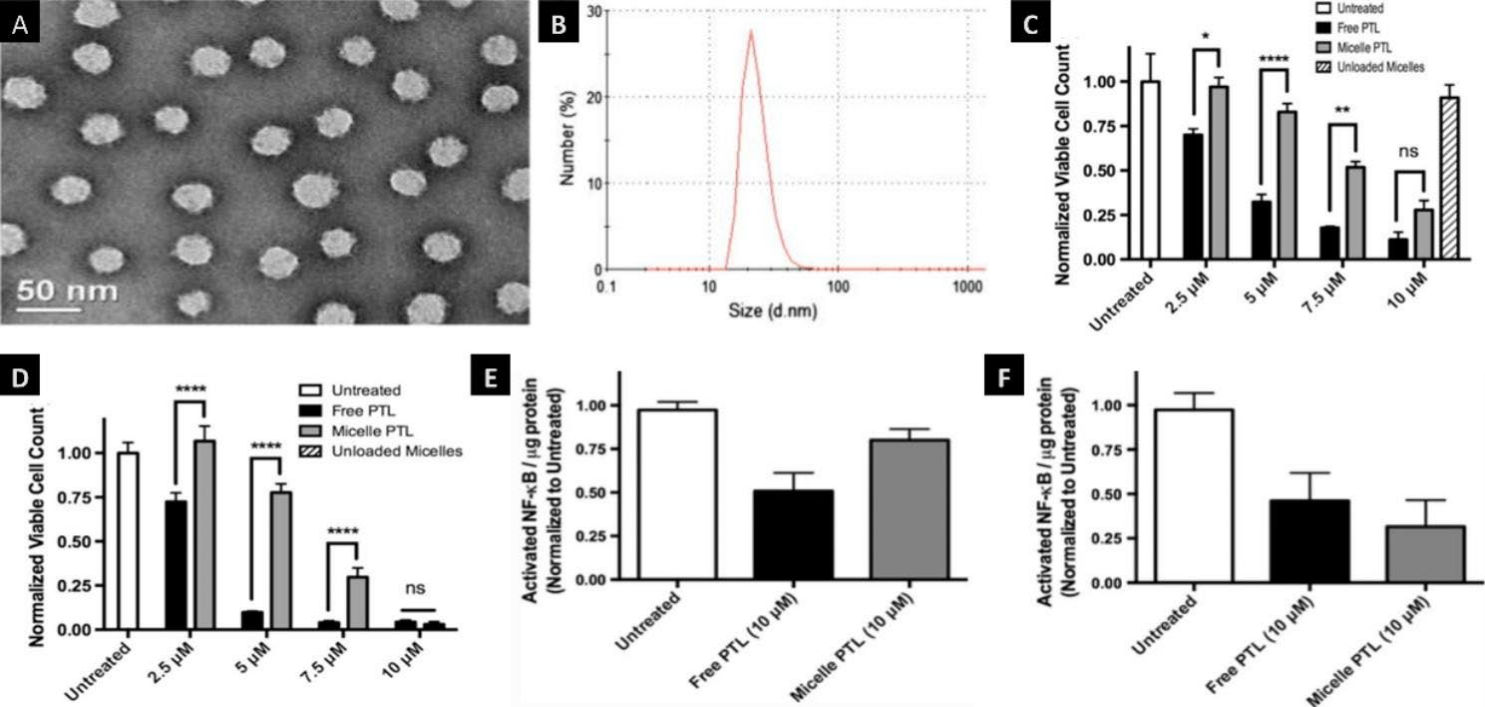
Nanomicelles delivery system of AML treatment **Note**: (**A-B**) Morphology and size diameters of the self-assembled nanomicelles (PTL-loaded-PSMA100-b-PS258). (**C-D**) In vitro cytotoxicity of PTL and PTL-loaded PSMA-b-PS micelles against MV4-11 cell after treatment for 24 and 48 h. (**E-F**) Activated NF-κB levels in MV4-11 cell nuclear extracts were quantified after treatment for 4 and 8 h by ELISA. The data is expressed as average ± S.D (n = 3). **P < 0.01,***P < 0.001.(Figure source from the reference [72], color figure can be viewed at springer.com.)

Nanomicelles with sizes in the 1-100 nm range are self-assembled, transparent colloidal dispersions composed of particles with a hydrophilic shell and a hydrophobic core. They are widely used in biomedical applications because of their moderate size, high solubility, and good stability, which make them versatile [62]. Nanomicelle is a self-assembled system formed by two surfactants, which consists of a hydrophilic head and a hydrophobic tail in a certain range of proportion; the hydrophobic core, through physicochemical interactions, can encapsulate hydrophobic drugs, while the hydrophilic tail contacts water to facilitate the solubility of hydrophobic drugs [67]. Moreover, the chemical structure can be transformed through surface modification and stimuli sensitization to make this system more stable. Compared to other common delivery systems, nanomicelles display enhanced tumor microenvironment accumulation through enhanced EPR effects. Lipids, amino acids, pluronics and acrylates are all classical polymer materials that form hydrophobic cores, while examples of polymers that form hydrophilic crowns include PEG, oxazoline, chitosan, dextran HPMA and so on [68]. Importantly, the behaviors of nanomicelles in vivo are determined by the high dilution process, the pressure generated by the injection, and the occurrence of serum and protein binding in the body [69]. For example, curcumin (CM), which is insoluble with poor bioavailability, is extracted from the turmeric rhizome and has been proven to be effective against leukemia through the mechanism of cell cycle retardation and inhibitory effects in FLT3-overexpressing AML cells [70]. Therefore, FLT3-specific curcumin-loaded nanomicelles were developed to improve the solubility and intracellular uptake. The uptake of the nanomicelles in leukemia cells was 2–3 times greater than that of free drug in DMSO [71]. The novel nanomicelles (PTL-loaded-PSMA100-b-PS258) with parthenolide (PTL) were developed. They found that the morphology and size diameters of these micelles have good properties in the Fig. 7A and B. They found that the it exhibited a dose-dependent cytotoxicity towards AML cells and were capable of reducing cell viability by 75% at 10 µM PTL, while free PTL were nontoxic at 24 h (Fig. 7C) and 48 h (Fig. 7D). The cytotoxicity of these micelles increased gradually over 24 h while free PTL achieved maximal cytotoxicity between 4 and 8 h (Fig. 7E-F), demonstrating micelle-mediated delivery of PTL to AML cells and stability of the micelles even in the presence of cells. And also, they found that free PTL and its micelles induced NF-κB inhibition at 10 µM PTL doses for treatment for 4 and 8 h (Fig. 7G-H), demonstrating some mechanistic similarities in cytotoxicity. Overall, this novel micelles remained stable over treatment times, gradually released PTL, and resulted in MV4-11 cytotoxicity and NF-κB inhibition [72].

## Regulation effect of nanomedicine properties

### Improved solubility and bioavailability

The good physiochemical properties of phytonanomedicines play a critical role in transmembrane permeability because they enable the drugs to cross intestinal biological membranes and enter the blood circulation. Nanotechnology can be applied to encapsulate phytomedicines to improve poor bioavailability [45, 73–75]. Some phytomedicines, such as pathenolide, have poor solubility, which also has adverse effects on bioavailability in vivo [76, 77]. Many studies have confirmed that wrapping nanoparticles or liposome on the surface of drugs and using special surfactants to form nanocrystals are effective ways to improve the solubility of drugs [78–80]. For example, wrapping PTL nanoparticles with PLGA and anti-CD44 could enhance their bioavailability to leukemia cells [59]. Therefore, it is important to increase solubility and improve the trans-membrane permeability of phytonanomedicines.

#### Facilitated absorption in pharmacokinetics

Nanomedicines are absorbed by macrophages, and lymphocytes are partially phagocytized and partially enter the circulatory system through capillary lymph vessels. Lian’s experiments confirmed that encapsulation with red blood cell membranes could protect the nanoparticles (RSANs) from being taken up by macrophages in vivo, which prevented the premature elimination of the drug [81]. Darwish et al. also found that PLGA-anti CD44-PTL-NPs were absorbed better than PLGA-PTL-NPs by leukemic cells. Moreover, wrapping PTL with PLGA and an anti-CD44 target antibody was an effective measure to promote uptake by leukemia cells [59]. Chung et al. found that functionalized Au-NPs promoted the cellular absorption of As_2_O_3_ in leukemia K562 and KA cell lines. Moreover, the cytotoxicity of for As_2_O_3_ to leukemia cells was greatly increased in the presence of Au NPs with dose-dependent inhibitionc.

#### Influenced distribution in pharmacokinetics

Nanomaterials related innovations in nanomedicine are flourishing because of the absorption, distribution, metabolism and excretion of nanoparticles in the body. Distribution is defined as the transport of nanoparticles and their drug carriers from blood to tissues, intercellular fluids, and cells. These medicines are absorbed and distributed throughout the body through the blood circulation. Peng’s experiments monitored the distribution of arsenic after intravenously administering FA-HSA-ATO to K562 tumor-bearing mice for 8 h. Moreover, the intratumoral arsenic concentration of the experimental group treated with FA-HSA-ATO was approximately 6-fold and 1.5-fold higher than those of the free ATO group and HSA-ATO group, respectively [82].

#### Effect transport in pharmacokinetics

Loading nanomedicine into nanomaterials can overcome obstacles to transport. For example, a special phosphatidylcholine nanoliposome was prepared to facilitate transport and penetration into leukemia cells by endocytosis or fusion processes [83]. J. Li et al. found that the efflux rate of BBR (berberine suspensions) was 3.07 ± 0.34, indicating the existence of efflux transporters (P-gp). Compared with that of a BBR suspension, the Papp of BBR nanoemulsion and its nanoemulsion + Ver was significantly reduced. This indicated that BBR efflux in the Caco-2 cell model can be alleviated by the use of a nanoemulsion. Moreover, the efflux of BBR was reduced under the action of the P-gp inhibitor verapamil (Ver), confirming that BBR is effluxed by P-gp [84]. Another experiment was conducted to examine the absorption of the berberine self-nanoemulsion system (BBR SNE). In the Caco-2 cell monolayer transport study (AP-BL), the absorption concentration of BBR suspension was almost undetectable. The efflux rate of BBR SNE was 2.47 ± 0.47, which indicated that efflux transporters may participate in this transport process. These data suggested that the ability of BBR-SNE to reduce BBR efflux in the Caco-2 cell monolayer may involve P-glycoprotein inhibitors [44].

#### Adjust metabolism in pharmacokinetics

Peng et al. found that compared to free ATO, FA-HSA-ATO had a markedly increased distribution half-life and elimination half-life. Thus, the increased circulation time in the body leads to arsenic accumulation in tumors, but accumulation is decreased significantly in various organs, such as the liver, heart, spleen, kidney and lung [82]. As seen in Hemeg’s experiments, although GA has well pharmacological efficacy against AML, its poor solubility and short half-life limit its clinical application [85]. Feng’s experiments showed that the AUC of the GA nanoemulsion was 3.18 times greater than that of the free drug. Additionally, the maximum concentration (Cmax) of the nanoemulsion was approximately 1.99 times higher than that of the free compound, and the mean residence time of the nanoemulsion was distinctly increased [43]. Similarly, in J. Li’s and Y. J. Li’s respective experiments, the AUC values of the BBR nanoformulations were much higher than that of the BBR suspension. Moreover, the MRT was also obviously elevated [44, 84]. In Wang Chuan’s in vitro dissolution test and in vivo bioavailability studies of As4S4, compared to the realgar raw material drug (R-As4S4), the water dispersibility and bioavailability of As_4_S_4_ in realgar water dispersal dosage form (E-As4S4) were both significantly improved [41].

### Reduced toxicity and side effects

Studies have shown that nanoemulsion alter the drug distribution in vivo. For example, acute toxicity results proved that the 50% lethal dose of the GA nanoemulsion (95% LD_50_, 21.7-25.16 mg/kg) was 1.26 times greater than that of its water solution (95% LD_50_, 16.84–20.53 mg/kg). Hematoxylin-eosin staining images suggested that the control group (blank nanoemulsion, BNE) had no obvious toxicity, while free GA had obvious toxicities to various important organs, including the liver, heart, lung, and kidney. The toxicity was reduced significantly, especially in the liver and kidney [43]. In in vitro cell experiments, e-AS4SA cocultured with the HL60 AML cell line effectively inhibited the proliferation of leukemia cells and the cytotoxicity of E-AS4SA was significantly higher than that of r-AS4SA. Therefore, various delivery systems, including nanomaterials with known structures, must be designed to efficiently deliver target drugs to target sites and reduce the distribution of free drugs in other tissues and organs [52].

### Avoid drug resistance

It is well known that drug resistance to treatment is the central barrier to the success of AML therapy, and the main influencing factor is the excessive expression of drug-resistant proteins such as P-gp encoded by MDR1 in cancer cells, which induces chemotherapy failure in patients. Nanotechnology can reverse drug resistance and improve the therapeutic efficiency of phytomedicines in a variety of ways, including enhancing the stability and solubility of drugs, regulating the drug release process, and improving drug targeting and transmembrane permeability. Guo et al.’s experiments found that the level of As_2_O_3_ in target leukemia KA cells was increased by the addition of Au nanoparticles (Au NPs). This suggests that Au NPs can inhibit the P-gp-mediated efflux of drugs from cancer cells, which helps to increase drug accumulation in targeted AML cells. As a result, Au NPs enhance the effect of As_2_O_3_ in obstructing the proliferation of KA cells, which are drug-resistant leukemia cells [86].

### Enhanced target

Since FLT3 was recognized as an AML treatment marker, the combination of targeted therapies with phytomedicine on the basis of nanotechnology approaches has been a research focus. For example, FLT3-specific curcumin-loaded polymeric micelles with incorporated cytokine receptors and EVQ peptides can improve the directional effect of curcumin micelles on FLT3 leukemia cells and exhibit significant cytotoxicity and inhibition of leukemia cells with FLT3 targets [66]. Vincristine (VCR) vinca alkaloid displays excellent anti-hematologic malignancy ability. However, it contributes less to AML therapy than other typical phytomedicines [87]. Its high toxicity and rapid PK behaviors restrict its application and reduce the amount of vincristine that reaches tumor sites. It was reported that CD44 is overexpressed on a majority of AML cells. KPSSPPEE(A6), as a urokinase-derived short peptide, exhibits high affinity for CD44[88]. Therefore, an A6-tagged cross-linked polymeric vincristine sulfate compound (A6-cPS-VCR) was formulated to treat CD44 + AML. Compared with normal VCR molecules, this novel compound displays definite targeting of CD44 + MV4-11 model leukemia cells, which contributes to enhancing its anti-leukemia influence in vitro and obviously improving the therapeutic efficacy in vivo [89].

### Prolonged release

Homoharringtonine (HHT) is a natural alkaloid that has been used to treat leukemia in our country for thirty years. However, it still presents problems such as fast release, insolubility and poor bioavailability. Many studies have used novel delivery systems, such as nanoparticles and advanced nanotechnology, to overcome these disadvantages. For example, Chen Meiyu reported that MNP-Fe_3_O_4_ nanoparticles delayed the release of HHT in vivo and improved the treatment efficiency against leukemia [58]. Similarly, it has been reported that a gambogic acid nanoemulsion could delay 90% drug release by 48, 12 and 18 times compared to its aqueous solution under different pH conditions (pH 2, pH 5.8 and pH 7.4)[48]. Another study showed that a self-nanoemulsifying system with berberine delayed the complete release 14.3-fold compared to its water suspension in PBS (pH 7.4). Moreover, the reason the novel system delays release is that the natural medicine is surface-absorbed or dissolved in the nanomaterials. An arsenic nanoparticle (2% drug loading, As-Ni PEG-PLGA) was released in different PBS solutions (pH 5.0 and 7.4). A rapid release of 20% of the arsenic from the nanoparticles occurred within 0.5 h at pH 7.4. Additionally, arsenic was burst released within 48 h by As-Ni, and the stable cumulative release rates were 43% (pH5.0) and 59% (pH 7.4). This result confirmed that these nanoparticles were very stable in neutral medium but sensitive to acidic conditions at pH 5.0[90]. In addition, while arsenic trioxide acid (ATO) underwent burst release within 2 h (98.61%) and was completely released within 4 h, the cumulative release rate of red blood cell membrane-camouflaged ATO-loaded sodium alginate nanoparticles was 95% within 84 h. These data suggest that these camouflaged nanoparticles have significantly sustained release compared to the nanoparticles in aqueous solution, as they form a physical barrier around the nanoparticles via RBCM that can significantly control drug release [81]. Similarly, other BSA nanoparticles containing AS_2_O_3_ were released rapidly within the first 2 h, with 75% of the drug released within 12 h; after that, the drug was released slowly within 24 h. The initial burst release may correspond to the surface adsorption of AS_2_O_3_, and the sustained release of the latter should be attributed to the incorporation of AS_2_O_3_ into the particle matrix, mainly owing to the erosion of the particle by the medium. Furthermore, there was an incomplete release (~ 5%), attributable to hydrophobic irreversible interactions of AS_2_O_3_ particles that were not subject to a decrease in albumin charge [81].

### Controlled delivery

**Fig. 8:**
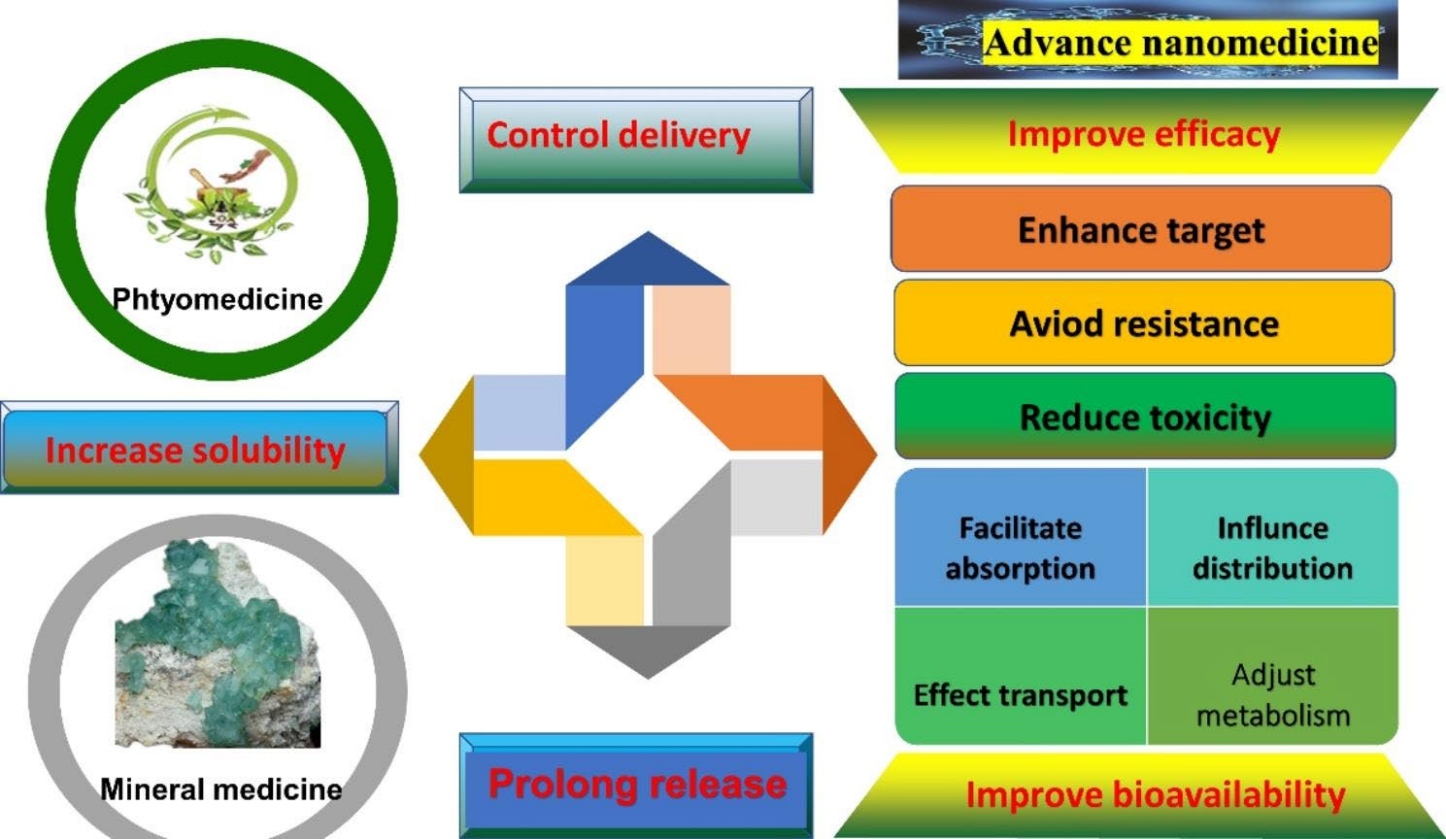
Regulation effect of nanomedicine of AML treatment **Note**: All in all, the regulation of nanomedicine is shown in the Fig. 8. We found that the novel nanomedicine drug delivery system can not only improve bioavailability through facilitating absorption, influencing distribution, effecting transport and adjusting metabolism, but also reduce toxicity and side effects. Important, it also avoids drug resistance, enhance target, prolong release and control deliver

Controlled delivery is an encouraging solution to some of the drawbacks of drugs, such as reduced solubility, limited biological distribution, tissue damage, rapid drug breakdown, cytotoxicity and side effects. A recently reported novel delivery system had a release curve that showed stability in a neutral environment (pH 7.4), releasing less than 20% arsenic. However, the release rate significantly increased and reached a plateau (75% arsenic) within 10 h under acidic conditions (pH 6.0). These results suggested that its delivery is controlled, and release occurs in an acidic environment [91] Arsenic trioxide acid was loaded onto folate (FA)-labeled serum albumin (HSA) pretreated with glutathione (GSH) to form a smart nanomedicine, FA-HSA-ATO, with release based on low pH and GSH-sensitive arsenic-sulfur bonds. The results showed that AS III-S release and control are susceptible to acidic or reductive environments. Then, the controlled drug release of this novel delivery system was slow (15% release within 24 h) in RPMI 1640 containing 10% FBS or human serum at pH 7.4. However, when the pH of the buffer was decreased or the GSH concentration was increased, the release of ATO from this delivery became rapid and eventually complete. ATO was released in the endosome, lysosome and cytosol after internalization. Controlled arsenic release was accompanied by a decrease in the size of this delivery system [82]. All in all, the regulation of nanomedicine is shown in the Fig. 8.We found that the novel nanomedicine drug delivery system can not only improve bioavailability through facilitating absorption, influencing distribution, effecting transport and adjusting metabolism, but also reduce toxicity and side effects. Important, it also avoid drug resistance, enhance target, prolong release and control deliver.

## Summary review and limitations

This review has concentrated on research on phytonanomedicines with novel delivery systems in recent years, including nanocrystal drug technology, superfine grinding technology, and other novel delivery systems, such as nanoemulsion, nanoparticles, nanoliposome, and nanomicelles. The table summarizes the data on the preparation and treatment response of different phytonanomedicine formulations **(**Table 1**).** Therefore, the foundational development of modern delivery systems and advanced combinations with phytomedicine components in AML treatment was reviewed, which could facilitate the further development of enhanced safety and efficacy in future research. Currently, standard chemotherapy can cure approximately 40–45% of young AML patients and cure approximately 10–20% of adult patients. Moreover, conventional chemical drugs usually have intolerable toxicity and easily lead to relapse or refractoriness after the development of resistance. Therefore, the cure rate of chemical treatment is less than 10%. Accordingly, gentler and more effective treatment is urgently needed. Phytomedicine, as a unique type of medical treatment, has been extensively used to treat various disorders in China for more than two thousand years. Many active ingredients of phytomedicines, such as arsenic trioxide and *Tripterygium wilfordii*, have pharmacological effects on AML [82, 92]. Based on modern concepts of pharmaceutical chemistry and pharmacology, the active components of phytomedicine have been separately characterized in terms of their regulatory effects on the signaling pathways of various tumor cells, which also have the potential for powerful effects on AML. The combination of phytomedicine and chemotherapeutic drugs can effectively treat leukemia and greatly reduce the side effects caused by chemotherapy. Such combinations also have the advantages of reducing drug resistance and decreasing adverse reactions. Increasing evidence demonstrates that multiple active components in phytomedicine therapeutic regimens with different but related mechanisms can enable maximal therapeutic efficacy with minimal adverse effects. However, due to the relatively complicated composition, uncertainty in the scientific evaluation systems for exact dosage and usage, and unclear relationships among pathways, it is essential to study the specific mechanisms of action of phytomedicines. In the future, the formulation, optimal dosages of active components of phytomedicine, and more effective drugs for AML treatment can be explored by combining the characteristics and clinical evaluation criteria, which could further improve patient survival. For example, the relationship between genomic features and drug responses was studied, focusing on the effects of 145 drugs, including chemotherapy drugs, natural components and targeted drugs, on thirty-three primary AML cell samples with a full genomic spectrum. These results showed that nanomedicine had nearly the same effects as the targeted medicine. More importantly, the researcher used network-based analysis to build a more precise prediction model than routine gene mutation data [81]. However, the clinical translatability of nanomedicine for AML therapy remains limited, especially for phytonanomedicine and mineral nanomedicine. Nowadays, there are few phytonanomedicine and mineral nanomedicine delivery system which have been approved for clinical therapy. From an industrial point of view, the scale-up of most reported nanomedicine from the bench scale to the mass production scale is complex because most of them have an intricate composition, are comprised of multiple components, and require multi-step preparation processes that are time-consuming and elevate the cost of their production [93–95]. To some extent, these make it difficult to promote nanomedicine in the clinical setting [96, 97]. Currently, microfluidic devices and 3D printing tools are the two most extensively utilized strategies for improving scalability of nanomedicine. These manufacturing process employs simple techniques for low-cost, readily scaled production [98]. Whilst there have been some efforts across academic communities and government agencies to form National Characterisation Laboratories, more explicit and stringent guidance is needed from the main governing bodies such as the FDA. Billions of dollars of investment have been funnelled into nanomedicine development over the past two decades, and unless there is clear leadership and guidance from the regulat-ory bodies, these efforts will not result in products coming to the market and future investment will be placed elsewhere [95].

**Table 1:** Overview nanomedcine per-clinical studies with design strategy and their advantages

Type	Drug	Materials	Size (nm)	PdI	Advantages	Ref.
Nanoparticles	arsenic trioxide	As-Ni PEG-PLGA coupled with DSS6 or F56	207.5-569.5	0.140–0.596	Target endosteal niche or vascular niche in the bone marrow; increase its loading capacity and controlled the drug release; be higher cytotoxic against K562 cells than free ATO and arsenic nanoparticles	[76]
		RBCM-SA	163.2 ± 4.4	0.27	Reduce the toxicity and improve the anti-tumor effects;. realize the safe, effective, and sustained release of ATO; remarkably improve drug safety during medical treatment.	[81]
		PEG-AS-RA	120.1 ± 72.3		Provide better biocompatibility and lower side effects;. be degraded by phospholipases to release As and RA contributing to therapeutic effect;. exhibit high water solubility and good biocompatibility; be highly stable in physiological buffers	[92]
		FA-HSA-ATO	43 ± 5.1		Recognize FRβ^+^ CML cells, resulting in more intracellular accumulation of ATO;upregulate FRβ expression,facilitating even more recruitment and uptake of FRβ-targeting drugs; alleviate side effects and improve therapeutic efficacy	[82]
		Au NPs	5		Increase the hydrophobicity of both cell suspensions; greatly decrease the peak potential of both cell lines; facilitate the cellular uptake of anticancer drug As_2_O_3_ into leukemia K562 cancer cells; inhibit the function of P-gp to improve the relevant drug accumulation in target drug-resistant cancer cells;enhance the cytotoxicity suppression of As_2_O_3_.	[86]
	HHT	HHT-MNP-Fe_3_O_4_	11.2		Enhanced inhibitory effect; induce more extensive apoptosis in leukemia cells; make more pronounced cell arrests at G_0_/G_1_ phase; reduce the expression of Mc-1 and activating caspase-3 and PARP	[54]
	GA + Fe_3_O_4_-MNP	Fe_3_O_4_-MNP			Promote GA-induced apoptosis in U937 cells;.The higher expression levels of caspase-3 and bax; down-regulate the expressions of bcl-2,NF-κB and survivin	
	curcumin	anti-CD123-Cur-NPs	181.27 ± 0.07	0.07 ± 0.03	Improve the ability of curcumin to induce apoptosis;achieve greater bioavailability; improved anti-cancer potential;bind to LSCs	[99]
	Partheno-lide	bone marrow directed multistage vector			MSV-PTL use approximately 40-fold lower dosage and 20-fold lower frequency than chemical analog of PTL (DMAPT) to cause the similar therapeutic effect; effectively release drugs to mouse BM enabling a low-bioavailability drug to kill AML cells; PTL was delivered in chemically intact form to the bone tissue; resulted in killing of LSCs; treatment with MSV-PTL indeed resulted in increased inhibition of NF-κB and elevated activation of γ-H2AX;facilitate delivery to the tumor niche	[100]
Solid lipid nanoparticles	berberine hydroch-loride	berberine hydrochlori	60.5			[101]
Nanoemulsion	GA	Tween-80, glycol, squalene	17.20 ± 0.11	0.198 ± 0.013	Stable; obviously delay release effect; Compared with its water solution, IC50 of the nanoemulsion were 1.67 times and 1.98 times higher and LD50 of it was 1.26 times higher;. enhance its poor solubility and safety; improved anti-tumour effect	[43]
	Berberine hydrochloride	Labrafil M 1944 CS, RH-40, glycerin, water			Relative bioavailability: 440.40%;significantly increase in intestinal permeability and reduce efflux of BBH by the multidrug efflux pump P-glycoprotein; improve stability, oral bioavailability and permeability	[84]
	berberine	RH40,1,2-propanediol, squalene	23.50 ± 1.67	0.121 ± 0.01	Slowly release; enhance oral bioavailability; enhance permeation and prevent efflux of BBR; extend survival time	[44]
Nanoparticles	GA and RACC	glycol chitosan nanoparticles	160 ± 4.86	0.14 ± 0.002	GA and RACC have the synergist effect; induced a remarkably higher apoptosis of cancer cells with ~ 28%;inhibits tumourigenicity and markedly suppress the cancer cell proliferation and metastasis; maintain the originality of GA without any degradation	[40]
	Partheno-lide	PLGA-antiCD44-PTL-NP	162	0.098	Improved the bioavailability and selective targeting of leukemic cells; improve the chance to target the leukemic cells and not harm normal cells.	[59]
	GA and magnetic nanoparticles of Fe3O4	MNPs-Fe_3_O_4_			Enhance obviously GA-induced cytotoxicity and apoptosis in K562 cells; MNPs-Fe3O4-drug delivery system can decrease the IC50 of GA and enhance apoptosis in leukemia cells;dramatically upregulated the transcription and expression of caspase-3 in K562 cells.	[102]
	arsenic trioxide	MnAs@SiO_2_-pHLIP	89 ± 4		Controlled release capacity; outstanding targeting ability	[103]
Nano-liposome	berberine hydrochloride	berberine hydrochloride liposomes	122 ± 3.5		Be interfered with glucose and lipid metabolism; prevent progression from hyperlipidemia to type 2 diabetes	[101]
	viblastine	Lip-C6 with viblastine			Overcome resistance to Lip-C6 by de novo AML;restore proapoptotic sphingolipid phenotype	[61]
	Arsenic trioxide	As (III)	100		Reduce the resultant acute toxic effect	[58]
	Ginseno-side	PM coating DOX co-loaded biomimetic nanosystem	100		PM naturally adheres to AML cell naturally adheres to AML cells;promote cytotoxicity;enhance ROS production; extend half-life of the nanosystem; significantly enhance ICD effect and ignite the immunity system;prolong the circulation time	[104]
Nanomicelle	curcumin	poloxamer-407,PBS-pH 7.4	35.6 ± 2.7	0.325 ± 0.038	Lengthen the half-life;higher cytotoxicity 3.2–3 times greater intracellular uptake to leukemic cells	[66]
	Partheno-lide	PSMA100-b-PS258	40 ± 10	1.05	Increase the aqueous solubility;extend release of PTL over 24 h; control drug-cell interactions and promote intracellular accumulation through clathrin-mediated endocytosis;robust PTL loading	[72]

## Future prospects

In addition to well-studied individual phytomedicine components, there are some empirical formulas composed of several useful components, such as the Shen Qi Sha Bai Decoction, that have proven to be effective in clinical application against AML, but the molecular mechanisms remain unknown. Network pharmacology was applied to demonstrate that this formula is effective in AML treatment, and the major candidate composition includes quercetin and licochalcone A [80]. Another successful formula, realgar natural indigo, is one of the conventional phytomedicine prescriptions in China for acute promyelocytic leukemia, which is a subtype of AML. The essential components include realgar and indigo, which have been proven to be effective against APL in vivo and in vitro. Xianxie Zhang et al. performed further studies on the realgar-indigo formula compatibility in bone marrow cells through the scRNA-seq method by analyzing transcriptome expression characteristics. This application of scRNA-seq to the study of phytomedicine formula theory broadened the perspective of how the combinations of these compounds influence the hematopoietic microenvironment. However, the limitations and further research based on this perspective can be summarized as follows: first, it is necessary to further elucidate the relationships between key genes in AML and phytomedicine compatibility by gene knockout and gene editing techniques, among others. Second, multiomics technologies, represented by single-cell scATAC-seq, could be used as powerful tools to study the mechanisms involving key genes in AML. Third, the current studies of AML disease are mainly based on mouse models. There are several important questions relating to phytonanomedicines, such as their safety profile, cost, chronic toxicity, large-scale manufacturing, and batch-to-batch variability. Further future research will be carried out in humans to examine clinical applications [105].

## Conclusion

With a long history of AML treatment development, nanomedicine including phytomedicine and mineral were found to enable significantly improved effects. Although these nanomedicines showed great therapeutic effects, their further clinical application is restricted by their water insolubility, adverse effects and serious toxicity. Secondary metabolites of phytomedicine and mineral, such as flavonoids, phenolic compounds, alkaloids, flavanols, terpenes, steroids, flavonols, organosulfur compounds and brassinosteroids, have been reported to destroy tumor cells by inducing cell cycle arrest and enhancing the apoptotic cell death process. Such agents with excellent anticancer efficacy, such as camptothecin, topotecan, irinotecan, etoposide,paclitaxel, docetaxel, vinblastine, and vincristine, have been used in various cancer treatments in the global market. To overcome their adverse effects, novel delivery systems based on nanotechnologies are being utilized. This review focuses on phytonanomedicine research involving nanocrystal, nanoemulsion, nanoparticles, nanoliposome, and nanomicelles drug delivery systems. Nanomedicine containing phytomedicine ingredients have been reported not only to improve the drug physicochemical characteristics, affecting the pharmacokinetics, increasing the stability, promoting the permeability, and improving the bioavailability, but also to show great therapeutic effects compared with free phytomedicine and mineral medicine, such as prolonged release, avoidance of drug resistance, reduced toxicity and enhanced targeting. This research may provide useful guidance for the use of phytomedicine constituents in AML treatment.

## Data Availability

The data used to support this review are included within the article.
